# Preliminary investigation of deoxyoligonucleotide binding to ribonuclease A using mass spectrometry: An attempt to develop a lab experience for undergraduates

**DOI:** 10.12688/f1000research.14268.2

**Published:** 2018-04-26

**Authors:** Daniel D. Clark

**Affiliations:** 1 Department of Chemistry and Biochemistry, California State University, Chico, Chico, CA, 95929-0210, USA

**Keywords:** education, biochemistry lab, protein-ligand interactions, mass spectrometry, ribonuclease A

## Abstract

Deoxyoligonucleotide binding to bovine pancreatic ribonuclease A (RNase A) was investigated using electrospray ionization ion-trap mass spectrometry (ESI-IT-MS). Deoxyoligonucleotides included CCCCC (dC _5_) and CCACC (dC _2_AC _2_).  This work was an attempt to develop a biochemistry lab experience that would introduce undergraduates to the use of mass spectrometry for the analysis of protein-ligand interactions.  Titration experiments were performed using a fixed RNase A concentration and variable deoxyoligonucleotide concentrations.  Samples at equilibrium were infused directly into the mass spectrometer under native conditions.  For each deoxyoligonucleotide, mass spectra showed one-to-one binding stoichiometry, with marked increases in the total ion abundance of ligand-bound RNase A complexes as a function of concentration, but the accurate determination of dC _5_ and dC _2_AC _2_ dissociation constants was problematic.

## Abbreviations

dC _5_                                 deoxyoligonucleotide with the sequence: CCCCC

dC _2_AC _2_                          deoxyoligonucleotide with the sequence: CCACC

RNase A                         bovine pancreatic ribonuclease A

ESI-IT-MS                      electrospray ionization ion-trap mass spectrometry

nESI-Q-TOF-MS           nanoelectrospray ionization quadrupole time-of-flight mass spectrometry

RNase A+dC _5_                ligand-bound form of RNase A (with one dC _5_ ligand)

RNase A+dC _2_AC _2_         ligand-bound form of RNase A (with one dC _2_AC _2_ ligand)

RSD                                relative standard deviation

## Introduction

Bovine pancreatic ribonuclease A (RNase A) is an endoribonuclease (EC 3.1.27.5) that hydrolyzes RNA. It is a small single chain polypeptide (124 amino acids) containing four disulfide bridges and is known for its significant stability ^1^. RNase A has been called “the most studied enzyme of the 20 ^th^ century” and it has seen wide use as a model protein in biochemical and biophysical experiments ^1^. Undergraduate life-science majors often learn of RNase A as part of a biochemistry course in the context of the Nobel Prize winning protein folding experiments performed by Christian Anfinsen ^2^. Students may also be familiar with the need to inhibit ribonucleases when working with RNA in the lab, often accomplished with diethyl pyrocarbonate, or will have learned about the role of ribonucleases in microRNA biology ^3^. Still others may recognize RNase A as an example of an enzyme that employs general acid-base catalysis as part of its chemical mechanism ^4^. Thus, RNase A is an excellent model for undergraduate lab experiments, not only because it has been extensively studied, but also because its use presents an opportunity to reemphasize important concepts in biochemistry and biology.

The application of mass spectrometry to the analysis of biomolecules has made an enormous impact in the life sciences. Protein identification, the characterization of protein modifications, and the quantification of biomolecules using mass spectrometry are commonplace. Of these, protein identification is the most established in an undergraduate teaching lab ^5– 10^. Numerous other biological applications of mass spectrometry have existed for many years, but some of these are arguably, less broadly appreciated, and this is especially true for undergraduates. Native mass spectrometry is an approach based on electrospray ionization, where biomolecules are sprayed from a non-denaturing solvent ^11^. Under such conditions, protein-ligand complexes can be maintained and a dissociation constant ( *K*_d_) can be determined via a titration experiment ^12– 14^. 

Previously, nanoelectrospray ionization quadrupole time-of-flight mass spectrometry (nESI-Q-TOF-MS) was used to investigate ligand binding to RNase A ^12, 15, 16^. These studies used nESI ionization for its superior sensitivity and relied on the TOF mass analyzer for its high mass range ^12, 15, 16^. In Zhang *et al.*, free RNase A and the ligand-bound forms of RNase A populated three charge states (+8, +7, and +6) at pH 6.6, with most of the signal (~90%) coming from the +7 charge state, which exceeded m/z 2000 in the ligand-bound forms ^12^. Similarity, in Sundqvist *et al.*, focus was placed on the +7 charge state of free RNase A and its ligand-bound forms ^15^. In contrast, Yin *et al.* reported the most abundant charge state of free and ligand-bound forms of RNase A to be +8 at pH 6.6 ^16^. Unfortunately, California State University-Chico does not own a nESI-Q-TOF-MS as employed by each of these research groups. Instead, we have an electrospray ionization ion-trap mass spectrometer (ESI-IT-MS), which by comparison to nESI-Q-TOF-MS, offers a lower sensitivity and mass range (50–2000 m/z). Consequently, at the outset of this preliminary investigation, it was recognized that observation of the +7 and +6 charge states of ligand-bound RNase A would not be possible with our instrument.

This work was an attempt to develop a biochemistry lab experience that would introduce undergraduate life-science majors to the use of mass spectrometry for the analysis of protein-ligand interactions. Two deoxyoligonucleotides, CCCCC (dC _5_) and CCACC (dC _2_AC _2_), were investigated for their ability to bind RNase A. Titration experiments were performed using a fixed RNase A concentration and variable deoxyoligonucleotide concentrations. Samples at equilibrium were infused directly into our ESI-IT-MS under native conditions. The relative simplicity of the sample preparation and instrument operation (by direct infusion) were viewed as desirable features for an undergraduate teaching lab. Data analysis was also straightforward. Herein is described the results of this preliminary investigation. This work differentiates itself from the abovementioned RNase A ligand binding studies (using mass spectrometry) by the experimental conditions employed, which includes the identity of the investigated ligands and the type of mass spectrometer used ^12, 15, 16^.

## Methods

### Sample preparation

A stock solution of bovine pancreatic ribonuclease A (#R6513, Sigma-Aldrich, St Louis, MO, USA) was prepared at 5.60 mg/mL in LC-MS grade water (Thermo-Fisher Scientific, Waltham, MA, USA). Ammonium acetate (NH _4_OAc) was LC-MS grade (#73594, Sigma-Aldrich). HPLC-purified deoxyoligonucleotides with the sequence “CCCCC” (dC _5_) and “CCACC” (dC _2_AC _2_) were obtained from ThermoFisher and the stock solutions (200 μM) were prepared in LC-MS grade water. Samples were prepared in 1.5 mL microcentrifuge tubes as indicated in Table 1. Six replicates were prepared and analyzed for “Sample 1” whereas “Samples 2–5” were prepared and analyzed in triplicate. Each sample was mixed by micropipetting, and incubated at room temperature for ten minutes, prior to analysis.

**Table 1.** Sample preparation.

Component	Sample #				
	1	2	3	4	5
RNase A (5.60 mg/mL) ^1^ (μL)	10	10	10	10	10
LC-MS grade H _2_O (μL)	40	37.5	35	30	20
20 mM NH _4_OAc, pH 6.00 (μL)	50	50	50	50	50
200 µM deoxyoligonucleotide ^2^ (μL)	0	2.5	5	10	20
Total Volume (μL)	100	100	100	100	100
Overall [deoxyoligonucleotide ^2^] (μM)	0	5	10	20	40
Overall [RNase A] (μM)	40.9	40.9	40.9	40.9	40.9

^1^409 μM RNase A; calculated with the MW _av_ (13,690.3) for PDB ID:1RTA (Ref. 17). ^2^Either dC _5_ or dC _2_AC _2_.

### Mass spectrometry

Samples were analyzed with a Thermo LCQ Advantage ion-trap mass spectrometer equipped with an electrospray ionization source. The instrument was operated in positive ion mode using a 4.5 kV spray voltage, 60°C capillary temperature, 200 ms inject time, 10 microscans, and nitrogen sheath and aux gas settings of 30 and 15, respectively. The instrument was tuned on the +8 charge state of free RNase A at m/z 1723.7 ( Table 2). Each sample was subjected to direct-infusion at 2.5 µL/min using the LCQ syringe pump and full-scan mass spectra (m/z 1500-1950) were collected for two minutes. The upper m/z range was capped at 1950 to exclude the +7 charge state of free RNase A, which in its various adduct forms, began at m/z 1955.5 ( Table 2). The rationale was that the +7 charge state of the ligand-bound forms of RNase A were above m/z 2000, which made +7 data incomplete and unusable ( Table 3). 

**Table 2.** Predicted m/z values for free RNase A with P _i_ adducts (X) ^1^. The +8 charge state used in this work is highlighted.

Ion	Free RNase A					
	X=0	X=1 P _i_	X=2 P _i_	X=3 P _i_	X=4 P _i_	X=5 P _i_
[M+X+H] ^+^	13682.3	13780.3	13878.3	13976.3	14074.3	14172.3
[M+X+2H] ^2+^	6841.7	6890.7	6939.7	6988.7	7037.7	7086.7
[M+X+3H] ^3+^	4561.4	4594.1	4626.8	4659.4	4692.1	4724.8
[M+X+4H] ^4+^	3421.3	3445.8	3470.3	3494.8	3519.3	3543.8
[M+X+5H] ^5+^	2737.3	2756.9	2776.5	2796.1	2815.7	2835.3
[M+X+6H] ^6+^	2281.2	2297.6	2313.9	2330.2	2346.6	2362.9
[M+X+7H] ^7+^	1955.5	1969.5	1983.5	1997.5	2011.5	2025.5
[M+X+8H] ^8+^	1711.2	1723.4	1735.7	1747.9	1760.2	1772.4
[M+X+9H] ^9+^	1521.2	1532.0	1542.9	1553.8	1564.7	1575.6
[M+X+10H] ^10+^	1369.1	1378.9	1388.7	1398.5	1408.3	1418.1

^1^Where X=0 (no phosphate adduct), X=1 P _i_ (+98), X=2 P _i_ (+196), X=3 P _i_ (+294), X=4 P _i_ (+392), X=5 P _i_ (+490).

**Table 3.** Predicted m/z values for the ligand-bound ^1^ forms of RNase A with P _i_ adducts (X) ^2^. The +8 charge state used in this work is highlighted.

Ion	RNase A+dC _5_						RNaseA+dC _2_AC _2_					
	X=0	X=1 P _i_	X=2 P _i_	X=3 P _i_	X=4 P _i_	X=5 P _i_	X=0	X=1 P _i_	X=2 P _i_	X=3 P _i_	X=4 P _i_	X=5 P _i_
[M+L+X+H] _+_	15066.2	15164.2	15262.2	15360.2	15458.2	15556.2	15090.3	15188.3	15286.3	15384.3	15482.3	15580.3
[M+L+X+2H] _2+_	7533.6	7582.6	7631.6	7680.6	7729.6	7778.6	7545.7	7594.7	7643.7	7692.7	7741.7	7790.7
[M+L+X+3H] _3+_	5022.7	5055.4	5088.1	5120.7	5153.4	5186.1	5030.8	5063.4	5096.1	5128.8	5161.4	5194.1
[M+L+X+4H] _4+_	3767.3	3791.8	3816.3	3840.8	3865.3	3889.8	3773.3	3797.8	3822.3	3846.8	3871.3	3895.8
[M+L+X+5H] _5+_	3014.1	3033.7	3053.3	3072.9	3092.5	3112.1	3018.9	3038.5	3058.1	3077.7	3097.3	3116.9
[M+L+X+6H] _6+_	2511.9	2528.2	2544.5	2560.9	2577.2	2593.5	2515.9	2532.2	2548.6	2564.9	2581.2	2597.6
[M+L+X+7H] _7+_	2153.2	2167.2	2181.2	2195.2	2209.2	2223.2	2156.6	2170.6	2184.6	2198.6	2212.6	2226.6
[M+L+X+8H] _8+_	1884.2	1896.4	1908.7	1920.9	1933.2	1945.4	1887.2	1899.4	1911.7	1923.9	1936.2	1948.4
[M+L+X+9H] _9+_	1674.9	1685.8	1696.7	1707.6	1718.5	1729.4	1677.6	1688.5	1699.4	1710.3	1721.2	1732.0
[M+L+X+10H] _10+_	1507.5	1517.3	1527.1	1536.9	1546.7	1556.5	1509.9	1519.7	1529.5	1539.3	1549.1	1558.9

^1^Where RNase A+dC _5_, L= +1383.9 (MW _av_) for one dC _5_, and RNase A+dC _2_AC _2_, L= +1408.0 (MW _av_) for one dC _2_AC _2_. ^2^Where X=0 (no P _i_ adduct), X=1 P _i_ (+98), X=2 P _i_ (+196), X=3 P _i_ (+294), X=4 P _i_ (+392), X=5 P _i_ (+490).

### Determination of total ion abundance

**Figure 1.**
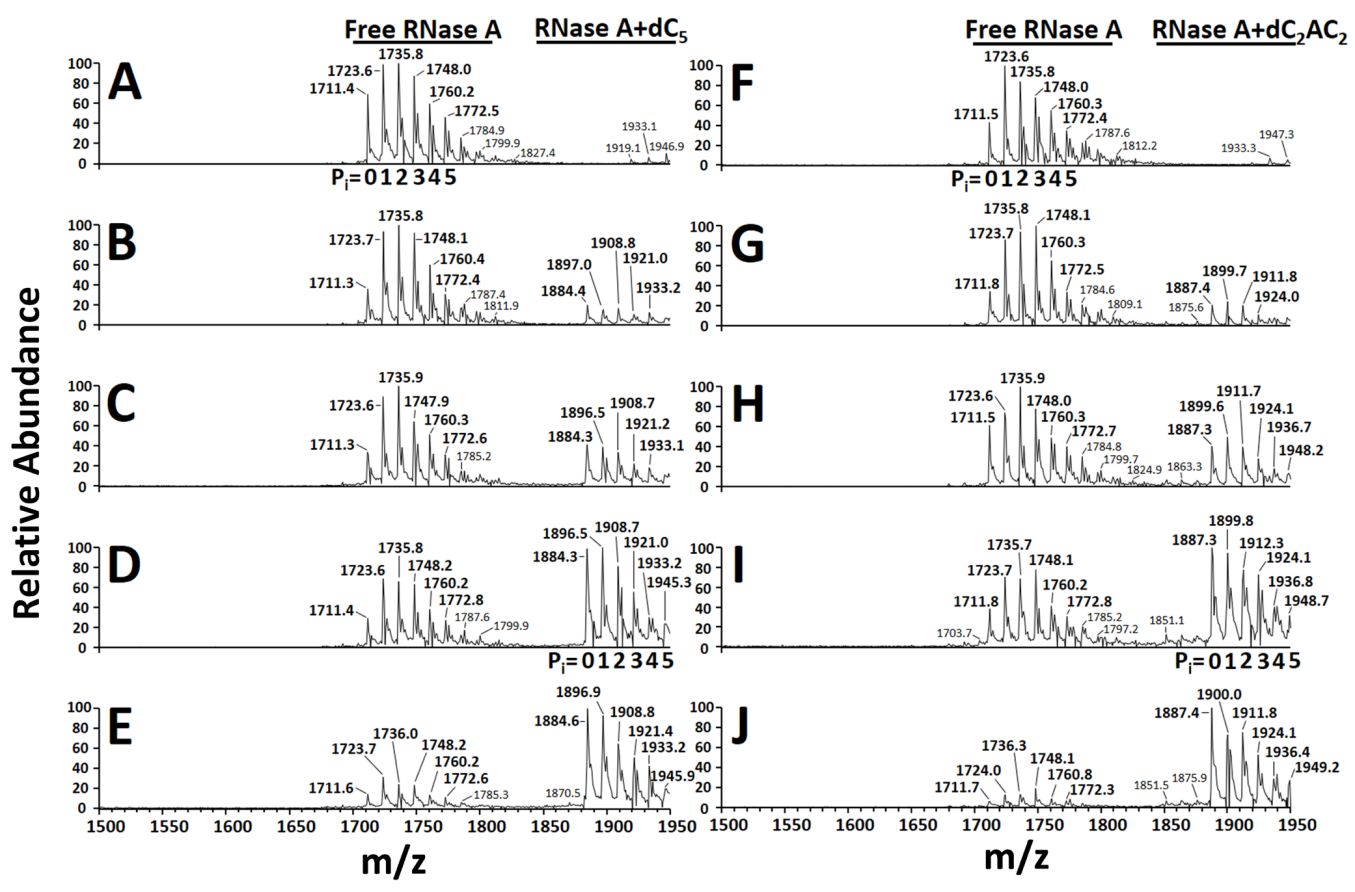
Mass spectra showing free RNAase A and ligand-bound forms as a function of added [deoxyoligonucleotide]. The +8 charge state is shown. ( **A** & **F**) no added deoxyoligonucleotide, ( **B**) 5 μM dC _5_, ( **C**) 10 μM dC _5_, ( **D**) 20 μM dC _5_, ( **E**) 40 μM dC _5_, ( **G**) 5 μM dC _2_AC _2_, ( **H**) 10 μM dC _2_AC _2_, ( **I**) 20 μM dC _2_AC _2_, and ( **J**) 40 μM dC _2_AC _2_. The number of phosphate adducts (P _i_= 0-5) are indicated in four representative mass spectra ( **A**, **D**, **F**, and **I**).

To facilitate determination of total ion abundance, tables of predicted m/z values for free RNase A ( Table 2) and the ligand-bound forms of RNase A (RNase A+dC _5_ and RNase A+dC _2_AC _2_) ( Table 3) were constructed. A series of 98 Da adducts were included in Table 2 and Table 3 due to their presence in the mass spectra of this work, and that of earlier studies ^12, 15^. These adducts have been suggested to be either H _2_SO _4_ or H _3_PO _4_^18^. Other RNase A studies have assigned these adducts as phosphate, and so each 98 Da adduct (X) in this work was designated as “P _i_” ( Table 2 and Table 3) ^12, 15^. Although mass spectra showed that free RNase A had up to 8 P _i _adducts ( Figure 1A and 1F), only the 0-5 P _i_ adduct forms of free RNase A and its ligand bound forms were used. This restraint was necessitated by the predicted m/z overlap of the ligand-bound forms of RNase A (with P _i_ adducts >5) with the m/z of free RNase at the +7 charge state. The “Qual Browser” feature of Xcalibur 1.4 SR1 software (Thermo) was used for analysis of each *.raw file. For each sample, mass spectra comprising the two-minute data collection were averaged. The “spectrum list view” was used to obtain intensity data for all of the ions in the ranges comprising the +8 charge state (with 0-5 P _i_ adducts) for free RNase A (m/z 1710.7-1772.9), RNase A+dC _5_ (m/z 1883.7-1945.9), and RNase A+dC _2_AC _2_ (m/z 1886.7-1948.9). The intensity data for all ions in each m/z range were added to give the “total ion abundance” of the free ( *Ab*_(P)_) and ligand-bound forms ( *Ab*_(PL)_) of RNase A. The total ion abundance for the ligand-bound forms (RNase A+dC _5_ and RNase A+dC _2_AC _2_) were plotted as a function of [deoxyoligonucleotide] using GraphPad Prism 7.

### Calculation of total ion abundance ratio and *K*_d_

The total ion abundance ratio was determined at each [deoxyoligonucleotide] using the method described by Kitova *et al.* ^13^, where for a 1:1 protein-ligand complex, the total ion abundance ratio ( *R*) is calculated using the total abundance of all ligand-bound ions ( *Ab*_(PL)_) and the total abundance of all free protein ions ( *Ab*_(P)_) as shown in Equation 1:

*R*= *Ab*_(PL)_/ *Ab*_(P)_ = [PL] _eq_/[P] _eq_          [1]

The total ion abundance ratio ( *R*) is used with the initial ligand concentration ([L] _0_) and initial protein concentration ([P] _0_) to calculate the association constant *(K*_a_) using Equation 2 ^13^:

*K*_a_= *R*/([L] _0_ − ((R/(1+R))[P] _0_))            [2]

The *K*_d_ can then be calculated as the reciprocal of the *K*_a_ value.

## Results

**Figure 2.**
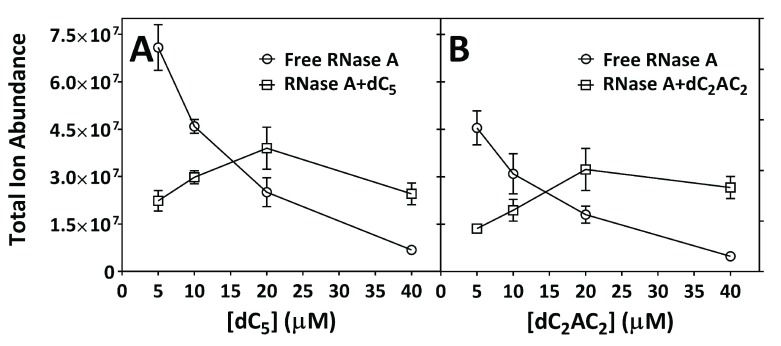
Total ion abundance for free RNase A and the ligand-bound forms vs. [deoxyoligonucleotide]. ( **A**) [dC _5_] and ( **B**) [dC _2_AC _2_]. The data is from Table 5, where points represent the average (n=3) ± standard deviation.

Table 1 indicates that samples contained an overall [RNase A] of 40.9 μM. Relatively low signal intensities observed for the +8 charge state of free and ligand-bound forms of RNase A necessitated this concentration, which was higher than the 5–20 μM RNase A used by others in nESI-Q-TOF-MS experiments ^12, 15, 16^. Table 2 and Table 3 present predicted m/z values for free RNase A and the ligand-bound forms of RNase A (RNase A+dC _5_ and RNase A+dC _2_AC _2_) with multiple P _i_ adducts, which correlated well with observed m/z values ( Figure 1). Upon increasing the concentration of dC _5_, the total ion abundance of free RNase A was found to decrease in intensity while the total ion abundance of RNase+dC _5_ was found to increase in intensity, which suggested 1:1 stoichiometry for the dC _5_:RNase A interaction ( Figure 1A–E). Similar results were seen for the titration using dC _2_AC _2_ ( Figure 1F–J). Table 4 presents total ion abundance data for free RNase A in samples that contained no added deoxyoligonucleotide. Total ion abundance data for free RNase A across six replicates gave a RSD of 16.4% ( Table 4). Table 5 contains total ion abundance data for free RNase A and the ligand-bound forms of RNase A in samples that contained various concentrations of dC _5_ or dC _2_AC _2_. Total ion abundance data across three replicates at each [deoxyoligonucleotide] exhibited RSD values of approximately 20% or less ( Table 5). A plot of the total ion abundance for free RNase A, RNase A+dC _5_, and RNase A+dC _2_AC _2_ as a function of [deoxyoligonucleotide] is shown in Figure 2. The total ion abundance for RNase A+dC _5_ and RNase A+dC _2_AC _2_ increased until 20 μM deoxyoligonucleotide, but decreased at 40 μM ( Figure 2). Table 6 presents the calculated total ion abundance ratio ( *R*) and dissociation constant ( *K*_d_) at each [deoxyoligonucleotide]. Samples containing <40 μM deoxyoligonucleotide unexpectedly produced negative *K*_d_ values ( Table 6). By contrast, Table 6 shows that samples containing 40 μM deoxyoligonucleotide produced consistent positive values where the average *K*_d_ for dC _5_ was 2.2 ± 0.1 μM and the average *K*_d_ for dC _2_AC _2_ was 1.0 ± 0.1 μM.

**Table 4.** Total ion abundance for free RNase A in samples that contained no added deoxyoligonucleotide. Data is for the +8 charge state.

Replicate	Free RNase A
**1**	71,438,882
**2**	80,188,529
**3**	70,622,004
**4**	94,929,471
**5**	61,169,836
**6**	65,198,871
**Average**	**73,924,599**
**SD**	12,135,483
**%RSD**	16.4

**Table 5.** Total ion abundance for free RNase A and the ligand-bound forms vs. [deoxyoligonucleotide]. Data is for the +8 charge state.

[dC _5_] (μM)	Replicate 1		Replicate 2		Replicate 3		Statistics					
	Free RNase A	RNase A +dC _5_	Free RNase A	RNase A +dC _5_	Free RNase A	RNase A +dC _5_	Free RNase A			RNase A+dC _5_		
							Average	SD	%RSD	Average	SD	%RSD
**5**	65,099,625	18,794,425	68,544,428	23,145,457	78,972,474	25,147,375	**70,872,176**	7,223,420	10.2	**22,362,419**	3,248,054	14.5
**10**	47,825,661	27,545,273	46,350,320	31,619,901	43,525,177	30,298,740	**45,900,386**	2,185,262	4.8	**29,821,305**	2,078,847	7.0
**20**	30,107,821	45,426,668	23,925,313	39,437,219	21,135,712	32,082,196	**25,056,282**	4,591,732	18.3	**38,982,028**	6,683,871	17.1
**40**	7,997,701	28,282,560	5,843,389	21,520,555	6,539,939	23,879,435	**6,793,676**	1,099,341	16.2	**24,560,850**	3,432,116	14.0
[dC _2_AC _2_] (μM)	Replicate 1		Replicate 2		Replicate 3		Statistics					
	Free RNase A	RNase A +dC _2_AC _2_	Free RNase A	RNase A +dC _2_AC _2_	Free RNase A	RNase A +dC _2_AC _2_	Free RNase A			RNase A+ dC _2_AC _2_		
							Average	SD	%RSD	Average	SD	%RSD
**5**	51,636,536	14,389,383	42,294,232	12,579,820	42,446,013	13,959,865	**45,458,927**	5,350,505	11.8	**13,643,023**	945,474	6.9
**10**	36,684,700	21,676,041	24,124,562	15,498,871	32,045,216	21,127,649	**30,951,493**	6,351,098	20.5	**19,434,187**	3,419,096	17.6
**20**	15,246,271	25,179,158	18,296,917	33,389,720	20,579,066	38,415,859	**18,040,751**	2,675,610	14.8	**32,328,246**	6,681,887	20.7
**40**	4,941,720	26,053,318	5,332,933	30,339,897	4,343,254	23,458,825	**4,872,636**	498,443	10.2	**26,617,347**	3,475,037	13.1

**Table 6.** The total ion abundance ratio ( *R*) and dissociation constant ( *K*_d_) calculated at each [deoxyoligonucleotide]. Data used for calculations was from Table 5.

[dC _5_] (μM)	Replicate 1		Replicate 2		Replicate 3	
	*R*	*K*_d_ for dC _5_ (μM)	*R*	*K*_d_ for dC _5_ (μM)	*R*	*K*_d_ for dC _5_ (μM)
**5**	0.29	-14.4	0.34	-15.8	0.32	-15.3
**10**	0.58	-8.6	0.68	-9.7	0.70	-9.7
**20**	1.51	-3.0	1.65	-3.3	1.52	-3.1
**40**	3.54	**2.3**	3.68	**2.1**	3.65	**2.2**
[dC _2_AC _2_] (μM)	Replicate 1		Replicate 2		Replicate 3	
	*R*	*K*_d_ for dC _2_AC _2_ (μM)	*R*	*K*_d_ for dC _2_AC _2_ (μM)	*R*	*K*_d_ for dC _2_AC _2_ (μM)
**5**	0.28	-14.0	0.30	-14.7	0.33	-15.6
**10**	0.59	-8.8	0.64	-9.3	0.66	-9.5
**20**	1.65	-3.3	1.82	-3.5	1.87	-3.6
**40**	5.27	**1.1**	5.69	**0.9**	5.40	**1.0**

LCQ *.raw data files for all samples Data files 1–6 are for samples that contained free RNase A (6 replicates), Data files 7–18 are for samples that contained RNase A and dC _5_ (3 replicates per [dC _5_]), Data files 19–30 are for samples that contained RNase A and dC _2_AC _2_ (3 replicates per [dC _2_AC _2_]). Click here for additional data file. Copyright: © 2018 Clark DD 2018 Data associated with the article are available under the terms of the Creative Commons Zero "No rights reserved" data waiver (CC0 1.0 Public domain dedication).

## Conclusions

This preliminary work demonstrates the potential and pitfalls of a LCQ ESI-IT-MS instrument to investigate protein-ligand interactions in an undergraduate teaching lab. Even though dC _5_ and dC _2_AC _2_ binding to RNase A are clearly illustrated in Figure 1, the presence of the P _i_ adducts complicated the mass spectra and broadened the signals for free RNase A and the ligand-bound forms of RNase A. In-source collision-induced dissociation was explored to reduce P _i_ adduct formation, but it appeared to disrupt the RNase A+dC _5_ and RNase A+dC _2_AC _2_ complexes, and so this approach was abandoned (data not shown). Although it was not attempted, centrifugal desalting of the RNase A stock solution might have eliminated P _i_ adducts and improved the quality of the mass spectra in Figure 1. As an added benefit, in the context of an undergraduate lab, desalting would also introduce students to a common sample preparation technique. It is unclear why the decrease in the total ion abundance for the ligand-bound forms of RNase A was observed at higher deoxyoligonucleotide concentrations ( Figure 2). Previously, the ion intensity ratio of free RNase A to the RNase A+cytidine 2′-monophosphate (2′-CMP) complex was observed to vary with charge state as follows: +8 (0.65), +7 (0.73), +6 (1.1) ^12^. This led Zhang *et al.* to suggest that either the binding of ligand, or the presence of ligand in the analyzed RNase A samples, created a change of the charge state distribution for the protein-ligand complex ^12^. In the present work, the binding of deoxyoligonucleotide, or the presence of deoxyoligonucleotide in samples, could have shifted some of the total ion abundance of free and/or ligand-bound RNase A from the +8 charge state to lower charge states, which were beyond the mass range of our ion-trap mass analyzer. This highlights an inherent limitation of this work, which was the inability to gather data for all free and ligand-bound RNase A charge states. Kitova *et al.* stated the importance of including all ligand-bound and free protein ions in the calculation of *R*, and emphasized that the “sometimes-used practice” of employing a particular charge state to determine *K*_a_ should be avoided ^13^. Thus, the lack of data for the +7 and +6 charge states of RNase A hindered accurate collection of total ion abundance data, which may have affected calculations of *R* and led to the negative *K*_d_ values at low ligand concentrations ( Table 6). Other factors to consider, that could have affected measurements, include non-ideal ionization conditions and non-specific ligand binding. Benkestock *et al.* showed that instrument-derived parameters (e.g. capillary-to-cone distances) could affect the protein-ligand complex to free protein ratio ^19^. They also demonstrated that compared to pneumatically assisted ESI, which was used in this work, nESI better reflects the equilibrium between free protein and protein–ligand complexes in solution. Furthermore, Kitova *et al.* noted that changes in the magnitude of Ka, with changes in ligand concentration, might indicate nonspecific ligand binding ^13^. As seen in Table 6, Kd values varied with the deoxyoligonucleotide concentration. Therefore, it is reasonable to suspect that non-specific binding may have contributed to the decreased total ion abundance of the ligand-bound forms of RNase A at higher ligand concentrations ( Figure 2). Notwithstanding these possibilities, the positive *K*_d_ values in Table 6 are of similar magnitude to those determined by Zhang *et al.* for 2′-CMP and CTP, via a nESI-Q-TOF-MS titration experiment, which were 1.7 ± 0.3 μM and 0.8 ± 0.2 μM, respectively ^12^. They are also in the neighborhood of in solution *K*_d_ measurements (3-24 μM) observed for the binding of short fluorescein-labeled deoxyoligonucleotides to RNase A ^20^. In conclusion, while RNase A is an excellent model for many experiments, instructors wishing to use a LCQ ESI-IT-MS instrument to investigate protein-ligand interactions are encouraged to consider other protein-ligand systems that would enable all charges states (of the free and ligand-bound protein) to be observed.

## Data availability

The data referenced by this article are under copyright with the following copyright statement: Copyright: © 2018 Clark DD

Data associated with the article are available under the terms of the Creative Commons Zero "No rights reserved" data waiver (CC0 1.0 Public domain dedication). 

Dataset 1. LCQ *.raw data files for all samples. 10.5256/f1000research.14268.d198373 ^21^

Data files 1–6 are for samples that contained free RNase A (6 replicates), Data files 7–18 are for samples that contained RNase A and dC _5_ (3 replicates per [dC _5_]), Data files 19–30 are for samples that contained RNase A and dC _2_AC _2_ (3 replicates per [dC _2_AC _2_]).
