# Supplementary material for: Preliminary investigation of deoxyoligonucleotide binding to ribonuclease A using mass spectrometry: An attempt to develop a lab experience for undergraduates
Source: F1000Res. 2018 Apr 26;7:340. Originally published 2018 Mar 20. [Version 2] doi: 10.12688/f1000research.14268.2 (PMC5897785; doi:10.12688/f1000research.14268.2)
Supplement: Peer review history

# Peer review: Referee response for version 2

**DOI:** 10.5256/f1000research.16067.r33468

**Published:** 2018-04-27

**Authors:** Samuel J. Allen

**Affiliations:** 1 BioElectron Technology Corporation, Mountain View, CA, USA

The revised report meets the suggested changes.

I have read this submission. I believe that I have an appropriate level of expertise to confirm that it is of an acceptable scientific standard.

# Peer review: Referee response for version 1

**DOI:** 10.5256/f1000research.15529.r32249

**Published:** 2018-04-12

**Authors:** Ryan N. Jackson

**Affiliations:** 1 Department of Chemistry and Biochemistry, Utah State University, Logan, UT, USA

In this manuscript, author Dan Clark describes an experimental protocol aimed at introducing undergraduate biochemistry students to mass spectrometry methods. Native electrospray ion-trap mass spectrometry (ESI-IT-MS) was used to produce mass spectra of unbound ribonuclease A (RNase A) and ligand – bound RNase A at differing concentrations of added oligonucleotides. Dissociation constants were determined using observed abundance ratios of bound and unbound RNAse A. However, dissociation constants with small concentrations of oligonucleotides were inaccurate, while the largest concentration produced a dissociation constant similar to that previously published. The author explains that these discrepancies likely result from limitations in the mass spectrometry equipment available, and concludes that a different protein – ligand combination may be better suited for the desired experiment.

 Overall this was a well-conducted investigation and meets an acceptable standard for publication. Contradictory to the conclusion of the author that another protein – ligand combination may be better suited to teach undergrads about mass spec, I found strong educational merit in the failures of this protocol to determine all dissociation constants. As an instructor of undergraduates I have found that students can often learn more when things do not work exactly as expected. The experiment presented here offers an opportunity for students to understand equipment limitations and may help students obtain a stronger understanding of how the diversity of charged states impacts the ability to collect an accurate mass spectra.

I have read this submission. I believe that I have an appropriate level of expertise to confirm that it is of an acceptable scientific standard.

# Peer review: Referee response for version 1

**DOI:** 10.5256/f1000research.15529.r32248

**Published:** 2018-04-09

**Authors:** Samuel J. Allen

**Affiliations:** 1 BioElectron Technology Corporation, Mountain View, CA, USA

**General Comments: **This work describes an experimental approach to introduce native mass spectrometry to undergraduate students. The author provides sufficient context for the choice of Ribonuclease A as the target protein, and describes the use of mass spectrometry under “native” conditions. This work also introduces undergraduates to practical use of mass spectrometry by providing “expected” m/z tables and shows the effect of adducted species on mass spectrometry signal. Plainly stated, the proposed experiment did not meet the initial hypothesis of the author. One major limitation to this study was the use of an ion trap mass spectrometer, which the author states as being the only available instrument at the academic institution. Additionally, the data from this study results in negative dissociation constant values for the lowest ligand concentrations, but reasonable dissociation constants at the highest ligand concentration. The author attempts to describe these results relative to other similar studies. As a result, this work describes the “potential and pitfalls” of attempting this experiment, which is useful for undergraduate students that are early in their scientific career.

**Suggestion ( no change requested):** For future investigations and undergraduate studies, it would be beneficial to use centrifugal desalting columns in an attempt to remove the phosphate adducts. This would result in improved ion response, less convoluted spectra, and would introduce undergraduate students to common sample preparation used in native-like protein MS experiments.

**Suggestion ( no change requested):** For future investigations and undergraduate studies, a native MS technique that has been used to address the upper m/z limitation of ion trap is to “supercharge” proteins ^1^. This can be done by adding as low as 1% v/v sulfolane or m-nitrobenzyl alcohol to the sample.

**Additional comments to conclusion ( minor revisions requested):** There are two issues that the authors addresses regarding the data from this study. (1) The observation of negative dissociation constants and (2) decreasing PL abundance at the highest L concentration. To point (1), although these experiments are being performed under native conditions (i.e. non denaturing solvents), there are still other factors during electrospray ionization that need to be considered during native experiments. For example, Benkestock, et al ^2^ show data that suggests that the “capillary-to-cone” distance and the electrospray probe internal diameter can affect the PL to L ratios. To point (2), the decrease in PL abundance at the highest L concentration may be due to non-specific binding as described in the already cited Kitova et al. (2012) ^3^(Section 2.4 and Figure 3). The author should add a couple sentences to the conclusion addressing how “non-ideal” ionization conditions and non-specific binding could have affected the measurements.

I have read this submission. I believe that I have an appropriate level of expertise to confirm that it is of an acceptable scientific standard.

## References

[1] GoingCCXiaZWilliamsER: New supercharging reagents produce highly charged protein ions in native mass spectrometry. *Analyst*.2015;140(21) : 10.1039/c5an01710f 7184-94 10.1039/c5an01710f 26421324PMC4617834

[2] BenkestockKSundqvistGEdlundPORoeraadeJ: Influence of droplet size, capillary-cone distance and selected instrumental parameters for the analysis of noncovalent protein-ligand complexes by nano-electrospray ionization mass spectrometry. *J Mass Spectrom*.2004;39(9) : 10.1002/jms.685 1059-67 10.1002/jms.685 15386746

[3] KitovaENEl-HawietASchnierPDKlassenJS: Reliable determinations of protein-ligand interactions by direct ESI-MS measurements. Are we there yet?. *J Am Soc Mass Spectrom*.2012;23(3) : 10.1007/s13361-011-0311-9 431-41 10.1007/s13361-011-0311-9 22270873
